# Sudden Unilateral Hearing Loss and Vertigo in an Adolescent: Viral Labyrinthitis Versus Idiopathic Sudden Sensorineural Hearing Loss

**DOI:** 10.7759/cureus.101445

**Published:** 2026-01-13

**Authors:** Victor Hugo Spitz, Jessica N Smock, Anna DeBonaventura, Kristie Rivers

**Affiliations:** 1 Medical School, Nova Southeastern University, Fort Lauderdale, USA; 2 Pediatrics, Broward Health Medical Center, Fort Lauderdale, USA

**Keywords:** autoimmune inner ear, cortical dysplasia, idiopathic sudden hearing loss, inner ear inflammation, labyrinthitis, pediatric vertigo, spiral ganglion injury, sudden sensorineural hearing loss, vestibular dysfunction, viral labyrinthitis

## Abstract

Labyrinthitis and idiopathic sudden sensorineural hearing loss (SSNHL) are uncommon in children and are both considered otologic emergencies, as delayed diagnosis can lead to permanent disability. We describe a 15-year-old boy with a history of focal cortical dysplasia and a single remote seizure who presented with abrupt left-sided hearing loss, tinnitus, vertigo, nausea, and headache. Neurologic examination revealed no focal deficits; however, marching Romberg testing demonstrated instability, and otoscopy revealed only a small left middle-ear effusion. Laboratory studies, including complete blood count and C-reactive protein, were unremarkable. Non-contrast computed tomography (CT) of the head showed no acute intracranial abnormality. Magnetic resonance imaging (MRI) of the brain and internal auditory canals with and without contrast demonstrated normal labyrinthine structures and stable right frontal cortical dysplasia. The working diagnosis, established by pediatric neurology and otolaryngology, was acute viral labyrinthitis or idiopathic SSNHL with vestibular involvement. The patient received high-dose systemic corticosteroids, vestibular suppressants, antiemetics, and empiric oral acyclovir. Vertigo and nausea improved substantially, but profound left-sided hearing loss persisted at discharge. Outpatient audiometry and possible intratympanic steroid therapy were planned.

This case emphasizes the diagnostic overlap between labyrinthitis and SSNHL in adolescents, the limited role of routine head CT for peripheral vestibulocochlear presentations, and the importance of early guideline-directed corticosteroid therapy. It also illustrates that MRI may be normal in clinically significant labyrinthitis and that careful follow-up is required to determine the need for salvage intratympanic steroid treatment.

## Introduction

Labyrinthitis is an inflammatory disorder of the membranous labyrinth that typically presents with acute-onset vertigo, imbalance, nausea, vomiting, and gait disturbance, usually accompanied by tinnitus and hearing loss [1]. Viral infections are the most common etiology, although bacterial labyrinthitis can occur following acute otitis media or bacterial meningitis and is associated with a higher risk of permanent cochleovestibular damage [1].

Idiopathic sudden sensorineural hearing loss (SSNHL) is generally defined as a sensorineural hearing loss of at least 30 dB over three contiguous frequencies developing within 72 hours in the absence of an identifiable cause [2]. Although rare in children, it should be considered whenever an otherwise healthy child presents with sudden unilateral hearing loss. Many patients, including pediatric cases, also report tinnitus and vertigo, which can blur the distinction between idiopathic SSNHL with vestibular involvement and viral labyrinthitis [3].

The 2019 American Academy of Otolaryngology-Head and Neck Surgery clinical practice guideline recommends prompt audiometric confirmation of SSNHL, early initiation of systemic corticosteroids, and consideration of intratympanic steroid injections as salvage therapy when recovery is incomplete two to six weeks after onset. The guideline advises against routinely prescribing antivirals, vasodilators, thrombolytics, or vasoactive agents [2]. When imaging is indicated, MRI of the brain and internal auditory canals and/or high-resolution temporal bone CT are preferred over routine non-contrast head CT, which has low diagnostic yield and provides limited visualization of the inner ear [2]. These guidelines also support intratympanic corticosteroid injection as salvage therapy for patients with incomplete recovery after systemic treatment, although practice patterns may vary regionally.

We report an adolescent with sudden unilateral profound hearing loss, tinnitus, and vertigo whose MRI was normal, highlighting the diagnostic and therapeutic challenges of differentiating viral labyrinthitis from idiopathic SSNHL in the pediatric population.

## Case presentation

History of present illness

A 15-year-old boy with a history of cortical dysplasia and a single non-febrile seizure at seven years of age presented to the emergency department with the sudden onset of left-sided hearing loss, tinnitus, vertigo, nausea, and headache. On the evening before the presentation, he noted decreased hearing and tinnitus in the left ear but was able to sleep. Upon awakening the next morning, he experienced complete hearing loss in the left ear with worsening tinnitus and new vertigo described as a spinning sensation, accompanied by nausea and diffuse headache. While en route to the hospital, he had one episode of non-bloody, non-bilious emesis. He denied recent upper respiratory infection, fever, otalgia, otorrhea, head trauma, barotrauma, recent air travel, or exposure to ototoxic medications.

Past medical history

At age seven, the patient experienced a prolonged seizure lasting approximately 45 minutes. Neuroimaging and neurologic evaluation at that time revealed cortical dysplasia in the posterior right frontal lobe. He was started on levetiracetam, which was discontinued after two years when insurance changed; his mother reported being told that he might eventually be able to stop the medication and tapered it herself. He has had no subsequent seizures. He reported a history of intermittent headaches responsive to acetaminophen or ibuprofen and no prior otologic disease, recurrent otitis media, or meningitis.

Examination

On arrival, he was afebrile and hemodynamically stable, appearing uncomfortable but nontoxic. The external auditory canals contained moderate cerumen bilaterally. Otoscopy revealed a small amount of middle-ear fluid behind the left tympanic membrane without erythema, bulging, or perforation; the right tympanic membrane appeared normal. Neurologic examination showed the patient to be alert and oriented with clear and fluent speech. Cranial nerves were intact with full extraocular movements, symmetric facial strength, midline palate elevation, and a midline tongue. Motor examination demonstrated normal bulk, tone, and 5/5 strength throughout without pronator drift. Sensory testing was intact to light touch and proprioception. Finger-to-nose testing and rapid alternating movements were normal. Marching Romberg testing demonstrated veering and instability, suggestive of vestibular imbalance, although gait was otherwise normal without truncal ataxia. No focal neurologic deficits were identified.

Laboratory studies

Initial laboratory evaluation included a comprehensive metabolic panel with sodium 139 mmol/L, potassium 4.5 mmol/L, chloride 107 mmol/L, bicarbonate 25 mmol/L, blood urea nitrogen 11 mg/dL, creatinine 0.7 mg/dL, and calcium 9.6 mg/dL. Liver function tests showed alanine aminotransferase 17 U/L, aspartate aminotransferase 35 U/L, and alkaline phosphatase 434 U/L. The alkaline phosphatase value is within the expected age-adjusted reference range for adolescents and likely reflects physiologic bone growth. Complete blood count demonstrated a white blood cell count of 7.15 × 10⁹/L, hemoglobin 14.5 g/dL, hematocrit 40.9%, and platelets 270 × 10⁹/L. C-reactive protein was less than 0.02 mg/dL. Overall, laboratory studies were unremarkable, with no leukocytosis or elevation in inflammatory markers. A summary of all laboratory investigations is provided in Table 1.

**Table 1:** Laboratory investigation results

Parameter	Patient Value	Reference Range
Sodium	139 mmol/L	135–145 mmol/L
Potassium	4.5 mmol/L	3.5–5.0 mmol/L
Chloride	107 mmol/L	98–107 mmol/L
Bicarbonate (CO₂)	25 mmol/L	22–29 mmol/L
Blood urea nitrogen	11 mg/dL	7–20 mg/dL
Creatinine	0.7 mg/dL	0.5–1.0 mg/dL
Calcium	9.6 mg/dL	8.5–10.5 mg/dL
AST	35 U/L	10–40 U/L
ALT	17 U/L	7–56 U/L
Alkaline phosphatase	434 U/L	Up to ~500 U/L (adolescent)
WBC	7.15 ×10⁹/L	4.5–11 ×10⁹/L
Hemoglobin	14.5 g/dL	13.5–17.5 g/dL
Hematocrit	40.9%	38–50%
Platelets	270 ×10⁹/L	150–400 ×10⁹/L
C-reactive protein	<0.02 mg/dL	<0.5 mg/dL

Imaging

**Figure 1:**
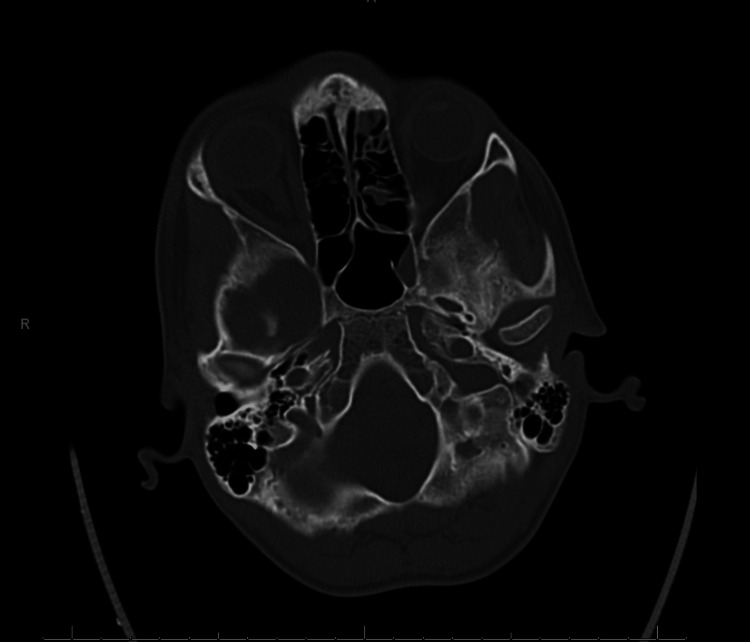
Non-contrast axial CT images of the head and temporal bones demonstrating no acute intracranial abnormality and normal-appearing internal auditory canals in this adolescent with sudden left-sided hearing loss and vertigo.

Non-contrast CT of the head was obtained in the emergency department and showed no acute intracranial abnormality or obvious temporal bone pathology. The temporal bones and internal auditory canals appeared normal (Figure 1).

**Figure 2:**
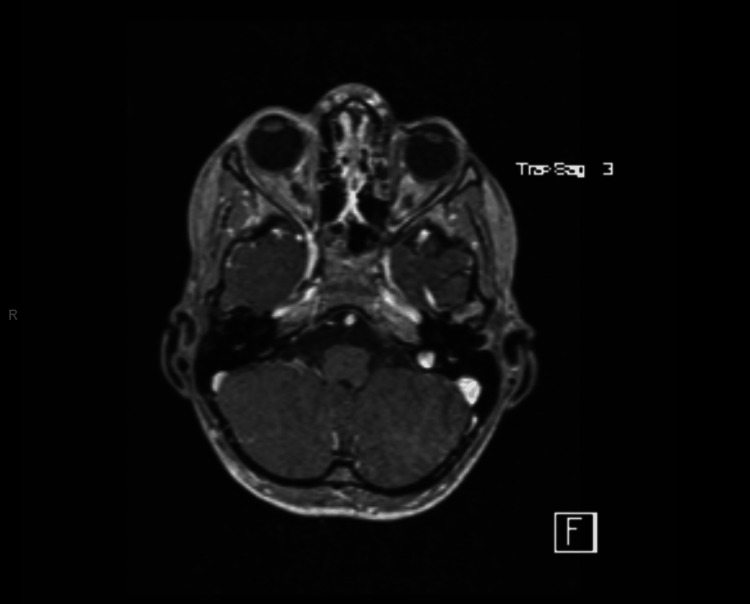
Axial T2-weighted MRI images of the posterior fossa and internal auditory canals showing normal labyrinthine structures without enhancement or mass lesion.

**Figure 3:**
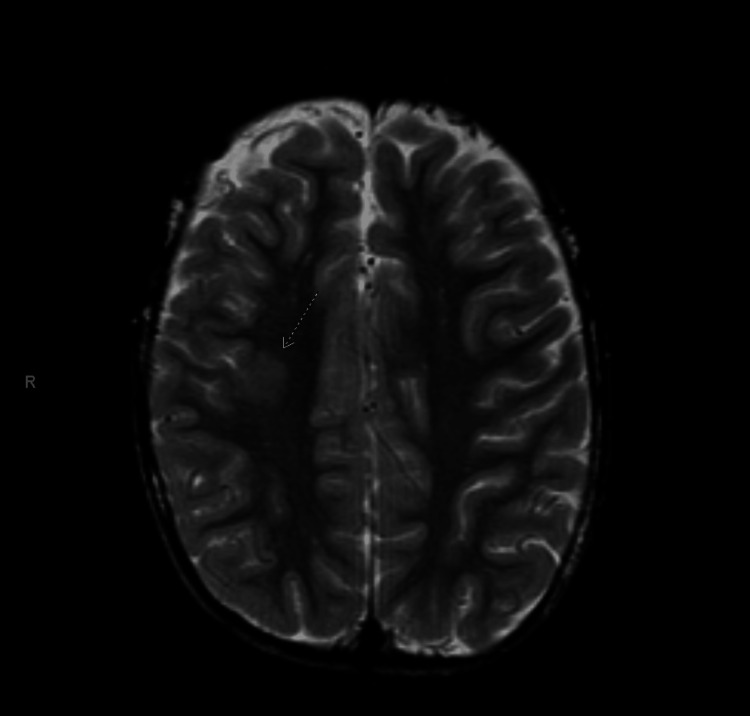
Axial T2-weighted MRI of the brain demonstrating asymmetric cortical thickening of the posterior right frontal lobe, indicated by the arrow, which is consistent with the patient’s previously diagnosed right frontal lobe cortical dysplasia.

Given the profound unilateral hearing loss with vertigo and the patient’s history of cortical dysplasia, pediatric neurology and otolaryngology recommended an MRI to rule out structural or anatomic pathology not visualized on CT. MRI of the brain and internal auditory canals with and without contrast demonstrated normal labyrinthine structures without enhancement and no mass lesion or infarct (Figure 2), as well as a stable area of asymmetric cortical thickening in the posterior right frontal lobe consistent with the known cortical dysplasia (Figure 3). No radiologic evidence of labyrinthitis or vestibular schwannoma was identified.

Hospital course and follow-up

The patient was admitted to a pediatric acute care unit for further management of presumed acute viral labyrinthitis versus idiopathic SSNHL with vestibular involvement. Pediatric neurology evaluated the patient and confirmed that the focal cortical dysplasia was a known, stable congenital finding without evidence of acute seizure activity or focal neurologic deficits; no electroencephalogram was indicated during this admission. Formal audiologic testing was not performed during hospitalization and was planned as an outpatient study to establish baseline severity and assess post-treatment recovery. Pediatric otolaryngology is considered a differential diagnosis that includes viral and bacterial labyrinthitis, idiopathic SSNHL, Ménière disease, vestibular schwannoma, and posterior circulation stroke. Bacterial labyrinthitis and central causes were felt to be unlikely in light of the normal inflammatory markers, absence of fever or meningitic signs, and reassuring MRI findings.

The patient received intravenous dexamethasone 10 mg every eight hours for three doses to reduce inner-ear inflammation, followed by a 12-day oral prednisone taper starting at 50 mg daily for seven days and then decreasing by 10 mg per day. Meclizine was prescribed for vertigo and ondansetron for nausea, with good symptomatic relief. Despite guideline recommendations against routine antiviral use in idiopathic SSNHL, oral acyclovir 800 mg three times daily for seven days was initiated empirically to cover possible herpes simplex virus involvement. Pantoprazole was given for gastrointestinal prophylaxis during high-dose steroid therapy.

Over the course of hospitalization, the patient’s vertigo and nausea improved substantially, and he was able to ambulate independently. However, he continued to report complete hearing loss in the left ear and mild residual headache. He remained afebrile and hemodynamically stable. He was discharged home after completion of the initial dexamethasone course with instructions to complete the prednisone taper and acyclovir regimen. Outpatient follow-up with pediatric otolaryngology was arranged approximately 10 days after discharge, with an additional follow-up visit with his pediatrician within two to three days. The plan included formal audiometry and consideration of intratympanic steroid injections if hearing recovery was incomplete within the recommended two-to-six-week window after symptom onset.

## Discussion

**Figure 4:**
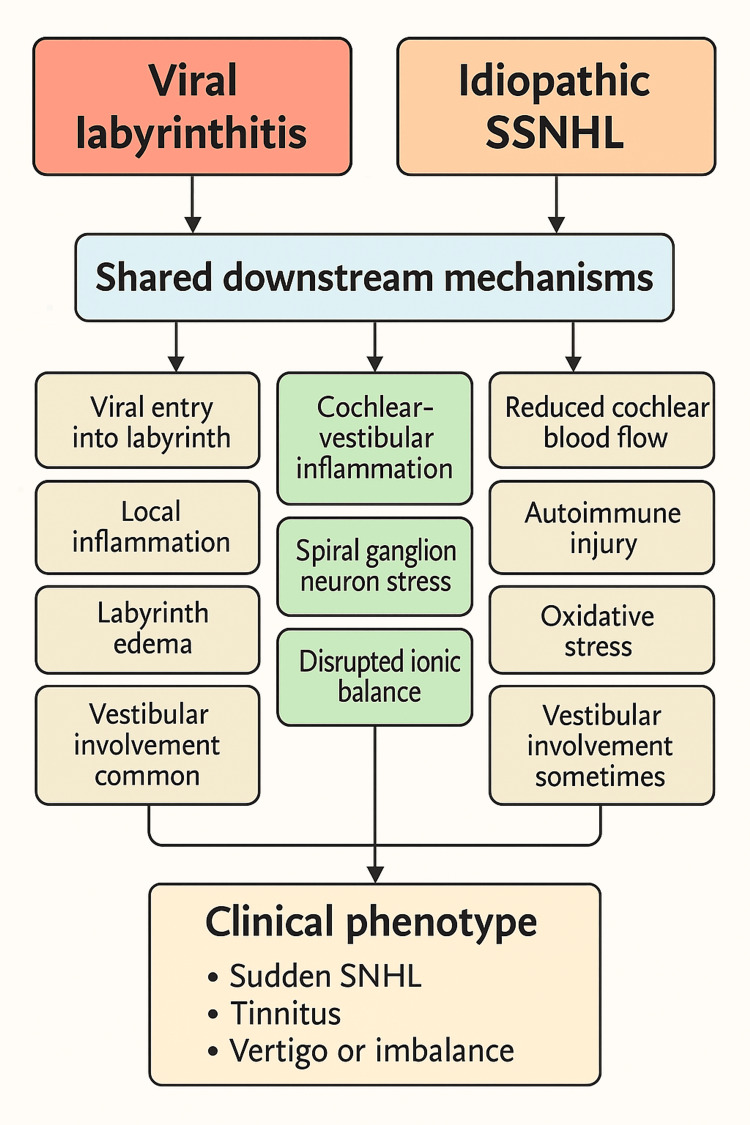
Conceptual model of the pathophysiologic mechanisms underlying sudden unilateral sensorineural hearing loss with vertigo. Schematic comparing viral labyrinthitis and idiopathic sudden sensorineural hearing loss (SSNHL), illustrating distinct upstream mechanisms and shared downstream cochleovestibular pathways producing a common clinical phenotype. Image created by the authors.

This case illustrates several important diagnostic and management issues in adolescents who present with sudden unilateral hearing loss and vertigo. Figure 4 provides a conceptual overview of the proposed pathophysiologic mechanisms linking viral labyrinthitis and idiopathic SSNHL.

The combination of acute profound hearing loss, tinnitus, and vertigo strongly suggests a peripheral cochleovestibular process [4]. The differential diagnosis includes viral or bacterial labyrinthitis, idiopathic SSNHL with vestibular involvement, Ménière disease, vestibular schwannoma, perilymph fistula, and posterior circulation stroke, among others [5].

In this patient, the absence of fever, otorrhea, or preceding otitis media and the presence of normal inflammatory markers made bacterial labyrinthitis unlikely. Ménière disease typically presents with recurrent episodes of vertigo associated with fluctuating, rather than sudden and complete, hearing loss and aural fullness. Vestibular schwannoma and other retrocochlear masses were effectively excluded by MRI of the brain and internal auditory canals. Posterior circulation stroke, particularly anterior inferior cerebellar artery infarction, can present with acute hearing loss and vertigo, but the patient lacked vascular risk factors or focal neurologic deficits, and MRI did not show evidence of infarction [5]. Although extended autoimmune or connective tissue serologies were not obtained during hospitalization, the absence of systemic symptoms, normal inflammatory markers, and the acute unilateral presentation supported classification as idiopathic sudden sensorineural hearing loss in this clinical context.

The two leading considerations were acute viral labyrinthitis and idiopathic SSNHL with vestibular involvement. Labyrinthitis is classically distinguished from vestibular neuritis by the presence of hearing loss in addition to vertigo [1]. Idiopathic SSNHL, however, frequently presents with tinnitus and vertigo, and some authors regard cochlear and vestibular involvement as a spectrum rather than distinct entities [6]. In our patient, the lack of a clear viral prodrome slightly favored idiopathic SSNHL, but the clinical overlap and similarity in recommended treatment mean that distinguishing between these diagnoses is often more academic than practical.

The imaging obtained in this case underscores the limitations of routine non-contrast head CT for peripheral vestibular presentations. Head CT is frequently ordered in emergency departments to evaluate dizziness or headache, yet it has a very low diagnostic yield for clinically significant findings and offers poor visualization of the posterior fossa and inner ear [7]. High-resolution temporal bone CT provides superior delineation of the bony labyrinth and is useful in conditions such as temporal bone fracture or superior semicircular canal dehiscence, while MRI of the brain and internal auditory canals is preferred for evaluating the membranous labyrinth, cranial nerves, and posterior circulation [8]. In our patient, CT was unrevealing, whereas MRI more directly assessed the regions of concern, although it remained normal with respect to the labyrinth.

MRI may be normal in early or mild viral labyrinthitis, and a lack of labyrinthine enhancement does not exclude the diagnosis. In such cases, the diagnosis remains primarily clinical, based on the acute onset of symptoms, lack of central neurologic signs, and recovery trajectory [9]. The present case, therefore, highlights the importance of correlating imaging with a careful neurologic examination rather than relying on imaging alone.

Systemic corticosteroids are widely accepted as first-line therapy for idiopathic SSNHL and are often extrapolated to presumed viral labyrinthitis, including in adolescents. Common regimens use oral prednisone at approximately 1 mg/kg/day (up to 60 mg daily) for 7-10 days, followed by a taper [2]. Our patient received high-dose dexamethasone followed by a 12-day prednisone taper starting at 50 mg daily, which is consistent with these recommendations. Evidence from adult studies suggests that earlier initiation of steroids is associated with better hearing outcomes, emphasizing the need for rapid recognition and treatment [2].

Intratympanic steroid injection offers the advantage of delivering high drug concentrations directly to the inner ear while minimizing systemic exposure. Randomized trials in adults indicate that intratympanic steroids are non-inferior to systemic steroids as primary therapy [10] and can provide additional benefit as salvage treatment for patients with incomplete recovery after systemic therapy alone [11]. Current guidelines, therefore, recommend offering intratympanic steroids as salvage therapy within two to six weeks of symptom onset when hearing recovery is insufficient [2]. In the present case, the plan for outpatient audiometry and possible intratympanic steroid therapy aligns with these recommendations.

The role of antiviral therapy in SSNHL and viral labyrinthitis remains controversial. Although herpes viruses have been implicated in some cases, randomized trials have not shown a clear benefit of adding antivirals to corticosteroids, and guidelines advise against their routine use [2]. Nonetheless, some clinicians still prescribe antivirals such as acyclovir empirically, especially in severe or complete hearing loss when the risk of adverse effects is low. This practice variation is reflected in our patient’s management and highlights the need for further pediatric-specific data.

Finally, this case underscores the importance of avoiding diagnostic anchoring on pre-existing structural brain lesions. The patient’s right frontal cortical dysplasia was a known congenital finding that could have distracted from a peripheral explanation for his new symptoms. However, careful neurologic examination and appropriate imaging demonstrated that the lesion was unchanged and unrelated to the acute presentation. Prognostic factors in sudden sensorineural hearing loss include the severity of hearing loss at onset, the presence of vertigo, and the timing of corticosteroid initiation, with earlier treatment generally associated with improved outcomes.

## Conclusions

An adolescent presenting with sudden unilateral hearing loss, tinnitus, and vertigo requires urgent evaluation for idiopathic SSNHL and labyrinthitis. This case highlights that MRI can be normal in clinically significant viral labyrinthitis, that routine head CT has limited value for peripheral vestibular presentations, and that early, guideline-directed corticosteroid therapy remains the cornerstone of management. Structured follow-up with audiometry is essential to assess recovery and determine the need for salvage intratympanic steroid injections. Further research is needed to optimize treatment strategies and clarify the role of adjunctive therapies, including antivirals, in pediatric patients.
